# Enhancing CYP3A4 Inhibition Prediction Using a Hybrid GNN–ML Model with Data Augmentation

**DOI:** 10.3390/ph19020258

**Published:** 2026-02-02

**Authors:** Somin Woo, Ju-Hyeok Jeon, Sangil Han, Changkyu Lee, Sang-Hyun Min

**Affiliations:** 1 Graduate School of Pharmacy, Kyungpook National University, Daegu 41566, Republic of Korea; jjunwm@gmail.com; 2 BK21 Healthcare Convergence Educational Group for Infectious Disease Management, Kyungpook National University, Daegu 41566, Republic of Korea; 3 Daegu RISE Center (Regional Innovation System & Education), Kyungpook National University, Daegu 41566, Republic of Korea; 4 GenON, Seoul 06285, Republic of Korea; wngur21011928@gmail.com; 5 Department of Innovative Pharmaceutical Sciences, Kyungpook National University, Daegu 41566, Republic of Korea; sangil.han@knu.ac.kr (S.H.); leeck30421@knu.ac.kr (C.L.)

**Keywords:** CYP3A4 inhibition, machine learning, graph neural network, SMILES augmentation, metabolic liability prediction

## Abstract

**Background/Objectives**: Cytochrome P450 3A4 (CYP3A4) metabolizes approximately 30–50% of clinically used drugs; thus, accurate prediction of CYP3A4 inhibition is essential for early assessment of drug–drug interaction (DDI) risk and toxicity. This study evaluated an integrated artificial intelligence framework for predicting CYP3A4 inhibition (%) using a large, curated chemical dataset. **Methods**: A dataset of 23,713 compounds was compiled from the Korea Chemical Bank and multiple commercial and public databases. Vector-based machine learning (ML) models (LightGBM, XGBoost, CatBoost, and a weighted ML ensemble) and graph neural network (GNN) models (O-GNN with contrastive learning and manifold mixup (O-GNN + CL + Mixup), D-MPNN, GINE, and GATv2) were evaluated. Manifold mixup was applied during GNN training, and SMILES enumeration-based test-time augmentation was used at inference. The best-performing ML and GNN models were integrated using a weighted ensemble strategy. Model interpretability was examined using SHAP analysis for ML models and occlusion sensitivity analysis for O-GNN + CL + Mixup. **Results**: The weighted ML ensemble achieved the best performance among ML models (RMSE = 19.1031, Pearson correlation coefficient (PCC) = 0.7566); the O-GNN + CL + Mixup model performed the best among GNN models (RMSE = 20.1002, PCC = 0.7265). The hybrid model achieved improved predictive accuracy (RMSE = 19.0784, PCC = 0.7570). External validation on 100 newly generated experimental data points confirmed generalizability (Custom Metric = 0.8035). **Conclusions**: This study demonstrated that integrating ML and GNN models with data augmentation strategies improves the robustness and interpretability of CYP3A4 inhibition prediction and established a practical framework for metabolic screening and DDI risk assessment.

## 1. Introduction

Cytochrome P450 3A4 (CYP3A4) is abundantly present in the liver and small intestine. It serves as a major drug-metabolizing enzyme in metabolizing approximately 30–50% of clinically used small-molecule drugs [1,2]. Inhibition of this enzyme is a major cause of drug–drug interactions (DDIs) and toxicity; therefore, evaluating a candidate compound’s metabolic stability and inhibition potential is an essential process in the early stages of drug development. Structural studies have indicated that CYP3A4 possesses a large active site volume and high structural flexibility, exhibiting changes in the binding conformations of diverse ligands [3,4]. This variability limits the prediction of the active site and quantification of the inhibition rates.

Traditional in vitro approaches (e.g., human liver microsomes and recombinant enzyme-based IC_50_/K_i_) offer advantages in standardization and interpretability but suffer from relatively high cost, time consumption, and limited scalability [5]. Meanwhile, docking-based structure prediction methods struggle with generalization to out-of-domain chemical spaces, partly due to the intrinsic flexibility of protein–ligand interactions. Various quantitative structure–activity relationship (QSAR)- and machine learning (ML)/deep learning-based CYP3A4 inhibitor prediction studies have been conducted [6,7,8,9,10]. For example, Veith et al. developed a model predicting the substrate/inhibitor status of multiple CYP enzymes [6]. Plonka et al. reported a CYPlebrity model to predict the inhibitors of several CYP enzymes [7]. Recently, deep learning-based approaches have been introduced, including Li et al.’s multitask autoencoder-based model [8], Wang et al.’s uncertainty-guided model [9], and Xiao et al.’s CYP inhibition prediction model [10].

However, most of these studies are designed around classification, limiting their ability to predict continuous inhibition strength (% inhibition or IC_50_). Certain issues still persist, including reduced generalization owing to differences in experimental conditions and data bias, along with insufficient explanatory power. In particular, considering the multiple binding modes and high structural diversity of CYP3A4, stable predictions in out-of-domain chemical spaces are difficult to achieve using a single model.

**Figure 1:**
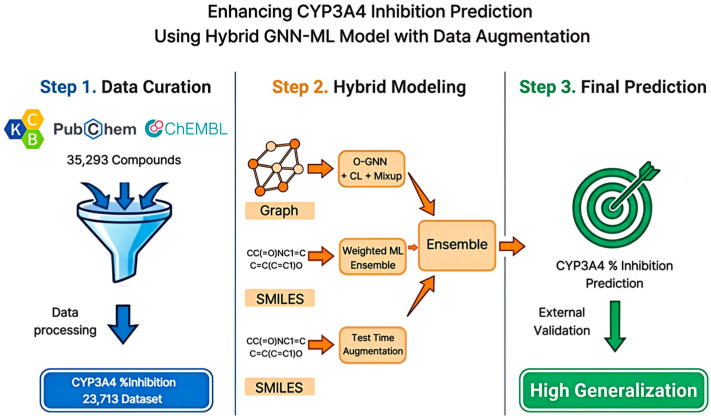
Schematic Overview of the Hybrid GNN-ML Framework for CYP3A4 Inhibition Prediction. The workflow includes data curation, hybrid modeling, and ensemble-based final prediction.

In this study, we integrated public data from the Korea Chemical Bank CYP3A4, (*n* = 1681) with other public databases to compile a dataset with 23,713 inhibition rate entries. Subsequently, a composite feature set, including a fingerprint, descriptor, embedding, and graph structure, was constructed. The generalization performance was enhanced by applying mixup augmentation and simplified molecular input-line entry system (SMILES)-based test-time augmentation (TTA). Furthermore, we compared and evaluated the ML-based methods (weighted ensemble, LightGBM, XGBoost, CatBoost) and graph neural network (GNN)-based methods [occlusion-based GNN (O-GNN) with contrastive learning and manifold mixup (O-GNN + CL + Mixup), directed message passing neural network (D-MPNN), graph isomorphism network with edges (GINE), and graph attention network v2 (GATv2)] (Figure 1). We identified key structural motifs contributing to CYP3A4 inhibition rate prediction using correlation analysis, SAR, and SHapley Additive exPlanations (SHAP) methods. This approach aims to rapidly exclude high-risk CYP3A4 inhibitor compounds in the early stages of drug development and manage development risks throughout the lifecycle.

## 2. Results and Discussion

### 2.1. Performance of the Proposed Model

In the ML model category, this study evaluated LightGBM [11], XGBoost [12], CatBoost [13], and a weighted ensemble model. O-GNN + CL + Mixup (O-GNN [14] model optimized with contrastive learning [15] and manifold mixup [16]), D-MPNN [17], GINE [18], and GATv2 [19] were evaluated in the GNN category. All models were trained and evaluated using identical data splitting and assessment protocols. Subsequently, the top-performing configuration from each category was selected for the final hybrid ensemble design.

In the ML category, the weighted ensemble achieved the best performance (root mean square error (RMSE) = 19.1031 and Pearson correlation coefficient (PCC) = 0.7566). In the GNN category, O-GNN + CL + Mixup achieved the best performance (RMSE = 20.1002, PCC = 0.7265). Based on these results, we constructed a hybrid ensemble system that integrates the complementary learning characteristics of the two categories. For this system, the weighted ensemble was selected as the ML component and O-GNN + CL + Mixup as the GNN component; SMILES-based TTA was additionally applied, and the ensemble weights were determined on the validation set. The final hybrid ensemble demonstrated excellent performance, achieving an RMSE of 19.0784 and a PCC of 0.7570 (Table 1).

ML models efficiently learn nonlinear interactions between physicochemical properties using global molecular descriptors, whereas GNNs directly capture local bonding patterns and interaction relationships within molecular graph structures, thereby exhibiting distinct inductive biases. This difference reduces the correlation between prediction errors. By combining the two model categories, the hybrid ensemble offsets bias and reduces variance, yielding a stable and consistent predictive performance.

### 2.2. Model Performance on the External Dataset

**Figure 2:**
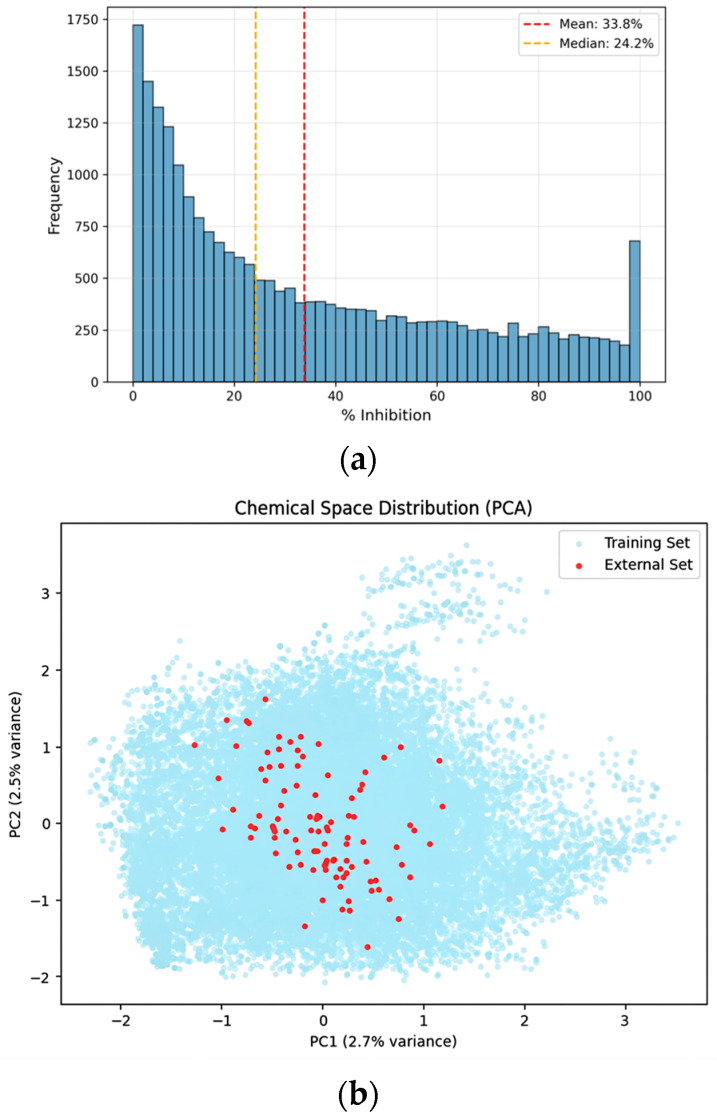
Distributional analysis and chemical space visualization of the curated dataset. (**a**) Distribution of the entire dataset: Histogram showing the frequency distribution of CYP3A4% inhibition values for the final curated dataset (*n* = 23,713). (**b**) PCA visualization: Principal component analysis (PCA) plot illustrating the chemical space of the training dataset (light blue) and 100 newly acquired external validation compounds (red). The projection indicates that the external compounds are distributed within the chemical space defined by the training data.

To validate the generalization performance of the developed model, an additional evaluation was conducted using an independent external dataset comprising 100 compounds that were not used at any stage of model training or validation. This external dataset represents a realistic secondary-screening scale following large-scale model development and was used exclusively for final performance assessment. To assess whether the newly acquired compounds occupy a chemical space comparable to that of the training set, principal component analysis (PCA), which preserves global variance, was applied for low-dimensional chemical space visualization (Figure 2). The PCA projection shows that the external validation compounds are distributed across the chemical space defined by the training data, without forming isolated or systematically shifted clusters. In this evaluation, the hybrid GNN-ML model outperformed both the standalone Weighted Ensemble and single O-GNN models, achieving a Custom Metric score of 0.8035. This demonstrates its robust predictive performance and improved generalization capability on previously unseen compounds.

### 2.3. Data Distribution and Heatmap of Collected Dataset

This heatmap displays the Spearman correlation coefficients between the CYP3A4 inhibition rate and key physicochemical descriptors. Red tiles indicate positive correlations, while blue tiles indicate negative correlations. Notably, Molar Refractivity, Molecular Weight (MW), and SLogP show moderate positive correlations with inhibitory activity, suggesting that molecular size and hydrophobicity are significant determinants of CYP3A4 inhibition.

**Figure 3:**
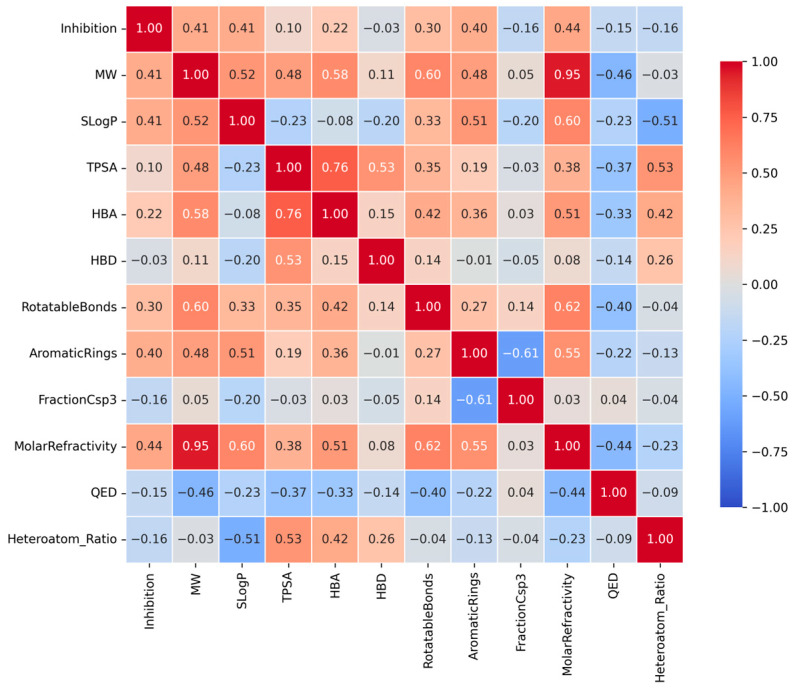
Correlation heatmap between CYP3A4 inhibition rates and key physicochemical properties.

Spearman’s rank correlation coefficients between CYP3A4 inhibition rate (%) and key physicochemical properties revealed weak positive correlations (Figure 3). Notably, Molar Refractivity showed the highest correlation coefficient, suggesting that molecular polarizability and size contribute to CYP3A4 inhibitory activity. Thus, molecules with high electron densities can form Van der Waals interactions within the hydrophobic pocket [3,4].

MW showed a significant positive correlation with inhibitory activity. Generally, CYP3A4 exhibits high affinity for large molecular weight ligands, consistent with the broad and flexible structure of the active site of the enzyme [3,4]. This trend aligns with the tendency of increased contact area and enhanced binding stability with increasing molecular volume.

Additionally, SLogP exhibited a positive correlation with inhibitory activity. This is consistent with the findings that increased molecular hydrophobicity enhances the affinity for CYP3A4’s hydrophobic binding pocket [3,4]. Molecules with higher LogP values demonstrated binding characteristics that were better suited to the nonpolar environment inside the enzyme, enabling efficient binding and inhibition.

Finally, the inhibition rate increased with the number of aromatic rings. Aromatic rings possess abundant electron density, enabling pi-pi stacking interactions with aromatic residues in CYP3A4 (e.g., Phe, Tyr, and Trp), which are key factors in enhancing binding stability [3,4,20]. CYP3A4 inhibitory activity was closely associated with molecular bulk, hydrophobicity, electron polarizability, and aromaticity. This trend reflects the properties of the active site, characterized by a large hydrophobic pocket and numerous aromatic residues, which are structural features of CYP3A4 [3,4] (Table 2).

### 2.4. Ring Substructure Type and Its Impact on Inhibitory Activity: A Structure–Activity Relationship

**Figure 4:**
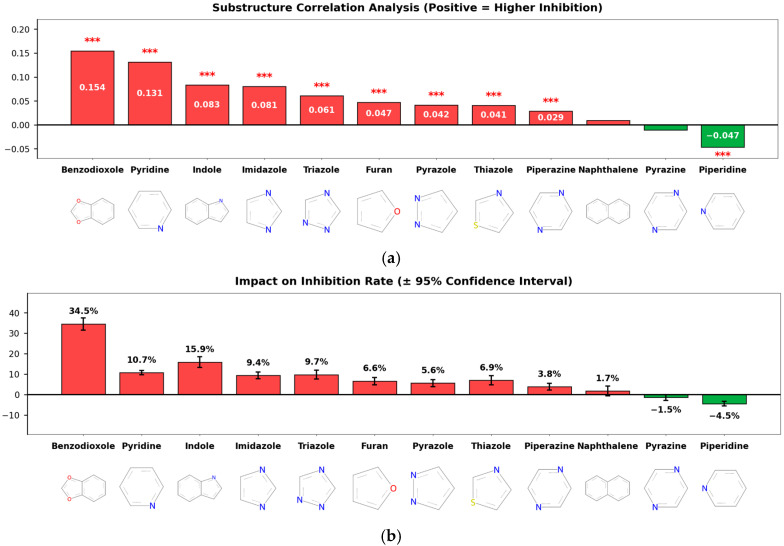
Substructure-Level Structure–Activity Relationship (SAR) Analysis. (**a**) Substructure Correlation: A bar chart showing the Point-Biserial correlation coefficients between specific ring substructures and CYP3A4 inhibition. Benzodioxole, pyridine, and indole rings exhibit significant positive correlations (red bars), whereas piperidine shows a negative correlation (green bar). Red asterisks (***) indicate statistical significance with *p* < 0.001. (**b**) Impact on Inhibition Rate: The mean difference in inhibition percentage between compounds containing vs. lacking specific substructures (error bars represent 95% confidence intervals). Compounds containing benzodioxole showed the highest increase in average inhibition (+34.5%), identifying it as a key pharmacophore for CYP3A4 inhibition.

This study aimed to analyze the SAR of inhibitory activity based on ring structure types, and accordingly, a substructure correlation analysis was performed (Figure 4a). The results showed that several heterocyclic and aromatic ring substructures, including benzodioxole (correlation = 0.154), pyridine (0.131), indole (0.083), and imidazole (0.081), exhibited statistically significant positive correlations with inhibitory activity [3,4], whereas piperidine showed a weak but significant negative correlation (correlation = −0.047).

Although the correlation coefficient of benzodioxole appears relatively modest, its magnitude should be interpreted in consideration of the limited prevalence of this substructure within the dataset. To quantitatively assess its practical effect size, Cohen’s d was calculated to evaluate the difference in inhibition rates between compounds with and without the benzodioxole scaffold. The resulting Cohen’s d value of 1.18 corresponds to a considerable effect size, indicating that the presence of the benzodioxole ring structure exerts a substantial and meaningful impact on CYP3A4 inhibition.

Analysis of the effect on average inhibition rate (Figure 4b) further supported these findings. Compounds containing benzodioxole exhibited the largest increase in mean inhibition, with an average increase of 34.5 percentage points. In addition, compounds containing indole and imidazole showed increases of 15.9 and 9.4 percentage points, respectively, while those containing pyridine also demonstrated an average increase of 10.7 percentage points. In contrast, compounds containing piperidine and pyrazine showed reductions in inhibition rates of 4.5 and 1.5 percentage points, respectively. These results indicate that specific ring substructures differentially influence both the direction and magnitude of CYP3A4 inhibitory activity.

The results further suggest that specific substructures and ring systems play important roles in inhibitory activity, providing useful information for identifying structural features that are recurrently associated with CYP3A4 inhibition [6,21].

The SAR analysis in this study focused on ring systems, aiming to capture structural signals that are stably observed in large-scale high-throughput screening (HTS) data environments. While substituent-level factors such as steric hindrance, heteroatom positioning, and electronic properties may also influence inhibitory activity, the present analysis emphasized the overall structure–activity trends driven by ring architectures rather than individual substituent effects.

### 2.5. Feature Importance in Machine Learning Model

ML-based feature importance analysis revealed that SLogP exhibited the highest importance, confirming that molecular hydrophobicity is a key determinant in predicting CYP3A4 inhibitory activity. This aligns with existing biochemical reports that CYP3A4 possesses a large hydrophobic binding pocket and forms strong van der Waals and pi-pi interactions with highly lipophilic ligands [3,4]. Consequently, molecules with high LogP values tend to exhibit enhanced inhibitory activity owing to the increased probability of interaction with the enzyme active site [20].

The second most important feature, MINdO, indicates the local electron density and topological environment of double-bonded oxygen atoms within the molecule. This implies that, beyond the mere presence of a functional group, the oxygen atom establishes a structurally favorable electronic environment that stabilizes binding by facilitating electrostatic interactions and hydrogen bond formation with the heme cofactor or surrounding residues within the CYP3A4 active site.

The FilterItLogS feature predicts the solubility of a molecule (log solubility), demonstrating that the balance between water and lipid solubility plays a crucial role in enzyme- binding efficiency. The active site of CYP3A4 is partially hydrophobic; however, molecules must maintain a certain level of water solubility to access the enzyme [5,6]. Therefore, molecules satisfying both the appropriate LogP and LogS ranges simultaneously form the most favorable structural profile for enzyme binding and inhibition.

The average similarity feature represents the structural similarity of each molecule to the other molecules within the training set. This acts as a variable that influences the prediction stability (reproducibility) and generalization performance during the model’s learning of structural patterns.

Additionally, carbon-centered features, such as MINssCH2, MAXaasC, MAXssCH2, and MAXaaCH, prominently reflect the length of the alkyl chain, the environment of the unsaturated bonds, and the electronic state of the aromatic carbons. These act as factors that determine the spatial fit and hydrophobic interaction strength of molecules within CYP3A4’s broad and flexible hydrophobic binding pocket [4]. Structures containing unsaturated carbons and aromatic rings particularly enhance binding stability by forming pi-pi stacking and non-covalent interactions with aromatic residues within the enzyme [3,4].

The properties of the VSA_EState and PEOE_VSA families reflect the surface charge distribution and the electronic state of a molecule. These metrics are calculated based on the partial charge and polarity of each atom, quantitatively describing the electrostatic interactions with the electrostatic environment within the enzyme pocket. CYP3A4 contains numerous polar residues, and this imbalance in charge distribution is a key factor in the enzyme–ligand binding affinity [3,4]. Therefore, the increased importance of these features demonstrates that the harmony between charge distribution and electron density plays a key role in determining the inhibitory activity.

**Figure 5:**
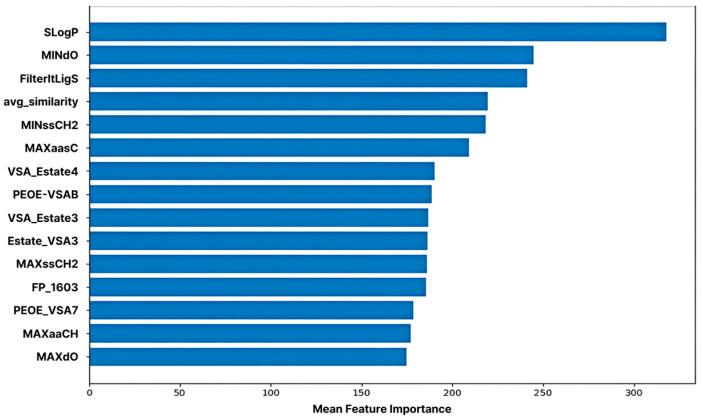
Feature Importance of the Machine Learning Model. The top 15 features are ranked based on their contribution to predicting CYP3A4 inhibition, as measured by the trained machine learning model.

Although the quantitative estimate of drug-likeness (QED) features did not rank among the top 15, they were positioned in the upper tier, indicating that drug-likeness is moderately associated with CYP3A4 inhibitory activity. QED comprehensively reflects various molecular properties, such as size, polarity, number of hydrogen bond donors and acceptors, and LogP [21]. Structures with better drug-like properties tended to exhibit greater enzyme-binding efficiencies (Figure 5).

Collectively, the feature importance analysis demonstrated that complex interactions between hydrophobicity, electronic properties, charge distribution, structural factors (alkyl chains and aromatic carbons), and drug-likeness play a critical role in predicting CYP3A4 inhibitory activity. These results align with the structural characteristics of CYP3A4 (a large lipophilic pocket and charge heterogeneity) and can serve as key structural descriptors for future enzyme–ligand interaction-based molecular design and inhibitor optimization studies [3,4].

The top 15 most influential features contributed to the model predictions. The log-partition coefficient (SLogP) was identified as the most critical predictor, followed by MINdO (electronic properties), and FilterItLogS (solubility). This ranking confirmed that lipophilicity, electronic interactions, and solubility balance were the primary factors governing CYP3A4 inhibitory potency.

### 2.6. SHAP Analysis in Machine Learning Model and Occlusion Sensitivity Analysis in the Deep Learning Model

**Figure 6:**
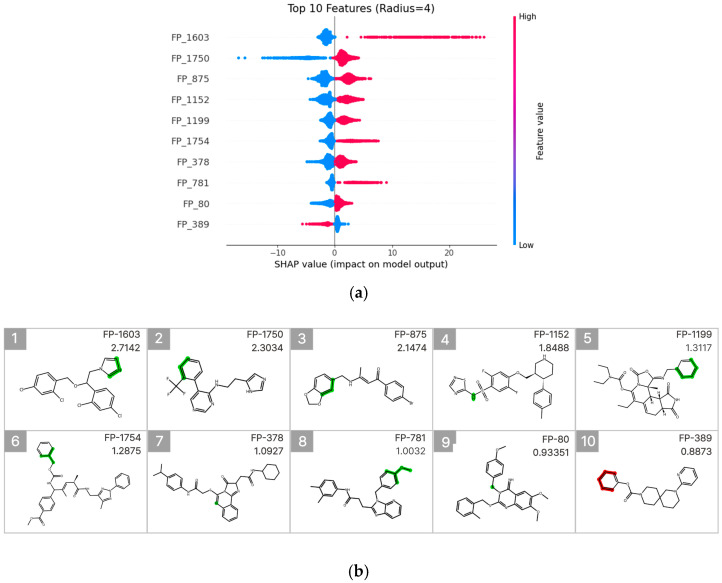
SHAP value analysis for model interpretation. (**a**) Summary plot ranking features by importance, with FP-579 showing the highest impact. Color indicates feature value (red: high, blue: low). (**b**) Molecular substructures of top 10 influential features with their SHAP values.

To interpret the prediction results of the molecular fingerprint–based LightGBM model, SHAP (SHapley Additive exPlanations) analysis was performed [22,23], and the results were visualized using a SHAP summary beeswarm plot (Figure 6a). To identify fingerprint (FP) features with high predictive importance, the top 10 FPs were selected based on their mean absolute SHAP values. For each FP, a representative molecule exhibiting both a high feature value and a high experimentally measured CYP3A4 inhibition rate was further visualized (Figure 6b).

Among these features, FP-1603 (mean absolute SHAP value: 2.7142) showed the highest contribution and was associated with a substructure containing an imidazole ring. This FP was predominantly concentrated in the high-feature-value region (pink) of the SHAP summary plot and exhibited a clear positive association with increased CYP3A4 inhibition. In contrast, FP-1750 (mean absolute SHAP value: 2.3034) displayed a broader distribution of SHAP values, suggesting that its contribution to inhibition prediction may depend on the surrounding molecular context. Additionally, FP-875 (mean absolute SHAP value: 1.1474) was associated with an aromatic substructure linked to a benzodioxole ring.

Furthermore, FP-1754 and FP-781 also exhibited relatively high mean absolute SHAP values and were identified as meaningful contributors to the model predictions. These features showed an overall trend of positive association with increased CYP3A4 inhibition in the SHAP summary plot. In particular, FP-781 was linked to an aromatic connecting structure containing an ether bond, which may contribute to modulation of molecular polarity and interaction potential with the CYP3A4 active site. In contrast, FP-389 exhibited predominantly negative SHAP values when present, indicating that the presence of this fingerprint is associated with a decreased likelihood of CYP3A4 inhibition. Collectively, these results suggest that CYP3A4 inhibition prediction is driven by a combination of diverse electronic and structural features rather than by a single functional group.

Although the final ensemble model, which incorporates high-dimensional embeddings, achieved high predictive performance, interpreting its internal decision-making process remains challenging owing to its structural complexity. To address this limitation and to visualize the learned SARs at the chemical level, two complementary interpretability strategies were employed. First, a LightGBM model using ECFP8 fingerprints [24] was adopted as a surrogate explainer for the weighted ensemble model, enabling visualization of substructure-level importance trends within the ensemble through SHAP analysis (Figure 6). Additionally, to directly interpret the prediction mechanism specific to the graph-based O-GNN + CL + Mixup model, an occlusion sensitivity analysis was performed by systematically masking atoms or substructures and evaluating the resulting changes in the predicted percent inhibition. This approach enabled model-specific identification of structurally relevant features that were effectively learned by the GNN.

**Figure 7:**
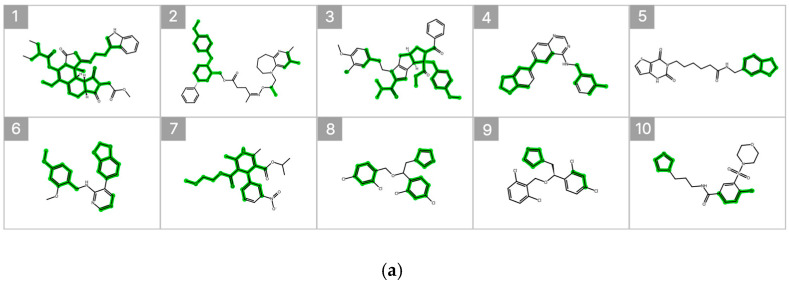

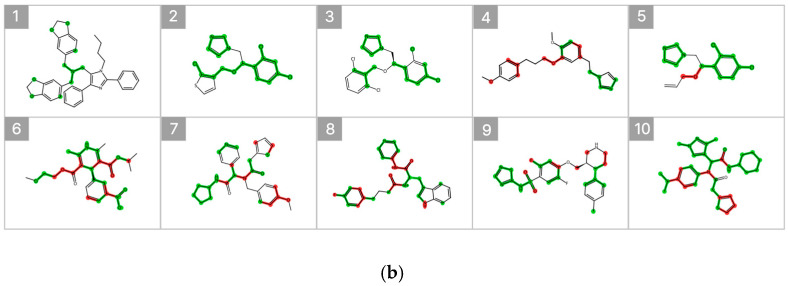
SHAP-Based Substructure Attribution in the Machine Learning Model and Occlusion Sensitivity Analysis in the Deep Learning Model. (**a**) SHAP-based substructure attribution for the top 10 compounds predicted to exhibit high inhibitory activity by the LightGBM surrogate model for the Weighted Ensemble. Green and red highlights indicate positive and negative contributions to the predicted percent inhibition, respectively. (**b**) Occlusion sensitivity analysis for the top 10 compounds predicted to exhibit high inhibitory activity by the O-GNN + CL + Mixup model. Green and red highlights indicate substructures whose masking leads to positive and negative changes in the predicted inhibition, respectively.

Figure 7 illustrates the substructure attribution results for the top 10 compounds predicted to exhibit high inhibitory activity. Substructures that contributed positively to the predicted percent inhibition are highlighted in green, whereas those with a negative contribution are indicated in red. The SHAP analysis revealed that specific heteroaromatic motifs, including benzodioxole, imidazole, and pyridine rings, consistently functioned as key contributors to increased inhibitory activity. These trends align well with the underlying data distribution and the established chemical intuition reported in previous SAR studies [6,20].

In addition, occlusion sensitivity analysis applied to the graph-based O-GNN + CL + Mixup model also identified benzodioxole-containing substructures among the top contributing features, and imidazole rings were frequently observed in compounds predicted to exhibit high inhibitory activity. These findings suggest that the weighted ensemble and O-GNN + CL + Mixup models capture chemically meaningful structural determinants that overlap partially but are emphasized from distinct perspectives. The complementary nature of these structural insights supports the integrated use of both models within the proposed framework.

## 3. Materials and Methods

### 3.1. Data Sources and Collection

The learning cohort for this study originated from 1681 CYP3A4% inhibition–structure pairs obtained from the Korea Chemical Bank (KCB) [25]. Furthermore, the external test data comprised 100 inhibition–structure pairs that were experimentally determined by KCB [2]. Although this dataset offered the advantage of consistency derived from a single institution and platform, its sample size limitations and bias toward specific molecular properties and scaffolds insufficiently represented the chemical space. Considering CYP3A4’s multi-binding mode characteristics, the generalizability of predictions based on such a small sample size was limited [3,4].

The CYP3A4% inhibition data used in this study were primarily generated from fluorescence- or luminescence-based HTS and quantitative HTS (qHTS) assays employing probe substrates such as Luciferin-IPA, midazolam, or testosterone. These assays quantify the reduction in CYP3A4 enzymatic activity in the presence of test compounds and report the results as percent inhibition values.

Owing to the nature of such screening assays, the dataset does not explicitly distinguish whether a compound acts as a CYP3A4 substrate or as a competitive or non-competitive inhibitor. Instead, it captures an inhibitory phenotype under standardized experimental conditions. In addition, % inhibition values measured at a single concentration (10 μM) were used; this is a commonly adopted strategy for large-scale screening and the comparative assessment of CYP3A4 inhibition liability.

To overcome these limitations, 33,612 external records were collected from ChEMBL [26] and PubChem [27]. (i) Only records explicitly describing CYP3A4% inhibition or activity measurements were included. (ii) Experiments solely dedicated to TDI (Time-Dependent Inhibition) were excluded. (iii) Results from a single 10 μM concentration were prioritized; for qHTS data, results most similar in concentration and conditions were selected. (iv) The species, matrix, and probe information were verified, retaining only entries that matched human CYP3A4. The collected 33,612 datasets were merged with 1681 datasets from the KCB to establish a total of 35,293 datasets.

### 3.2. Data Processing

To preprocess the collected dataset of 35,293 entries, we performed desalting and solvent removal followed by canonicalization, which involved removing physically implausible values (below 0% or above 100%) or clipping them within the valid range.

Duplicate structures were merged into a unified standard Canonical SMILES format. To account for experimental variability in CYP3A4% inhibition measurements, the label aggregation strategy for duplicate SMILES was extended beyond simple averaging to a piecewise aggregation scheme based on inhibition intervals. For compounds with repeated measurements, the average inhibition rate was first calculated. If this average was below 20%, the minimum value was adopted as the representative label to prevent false positives arising from a small number of spuriously high measurements. Conversely, when the average inhibition exceeded 70%, the maximum value was selected to preserve strong inhibition signals that might otherwise be diluted through averaging and misclassified as false negatives. For intermediate inhibition values (20–70%), the arithmetic mean was used, as variability among replicate measurements was generally stable and representative.

To evaluate the robustness of the selected thresholds (20% and 70%), a sensitivity analysis was conducted by systematically varying the lower threshold between 10 and 30% and the upper threshold between 60 and 80. For each threshold combination, both the total number of retained data points and the mean percent inhibition value were examined. Across all tested threshold settings, the dataset size remained unchanged, and variations in the mean inhibition rate were minimal (within 0.07%), indicating that the aggregation results were not overly sensitive to the specific threshold selection.

Based on this robustness assessment, 20% and 70% were adopted as conservative and stable thresholds. Inhibition values below 20% are more susceptible to experimental variability or non-specific responses in CYP3A4 activity assays, whereas values above 70% reflect a substantial reduction in enzymatic activity and enable clear discrimination of strongly inhibitory compounds.

Furthermore, compounds exhibiting excessively large deviations among replicate measurements were considered outliers, indicating reduced reproducibility due to differences in experimental conditions or batch effects [28]. Entries meeting predefined exclusion criteria (e.g., probe–matrix mismatch or unrealistic deviations) were excluded. This interval-based aggregation and outlier exclusion strategy was designed to mitigate floor/ceiling effects and repeatability issues commonly observed in single-concentration HTS data. Through this refinement process, a curated CYP3A4% inhibition dataset comprising 23,713 compounds was established.

To further assess the chemical diversity of the final curated dataset, Bemis–Murcko scaffold analysis was performed. Consequently, 13,241 unique scaffolds were identified from 23,713 compounds.

### 3.3. Metrics

Model performance was evaluated using standard regression metrics, including RMSE, coefficient of determination (R^2^), and PCC, to collectively assess absolute prediction accuracy and linear correlation between predicted and observed values (Equations (1)–(3)).

Furthermore, a custom composite metric was defined to provide a balanced evaluation tailored to CYP3A4% inhibition prediction. This metric could simultaneously capture absolute prediction accuracy and relative inhibition trends across compounds. Specifically, it could integrate a normalized error-based component, which assigns higher scores to smaller prediction errors, with the PCC, which evaluates the preservation of relative inhibition patterns and ranking among compounds. Both components were combined with equal weights in the composite score (Equation (4)).

By combining these complementary metrics, the proposed Custom Metric enables robust and numerically accurate evaluation, as well as allows models to consistently capture relative changes in inhibition values. Equal weighting (0.5/0.5) was selected as a neutral choice, as variations in the weighting scheme affected the absolute score values but did not alter the relative performance trends among models. This metric aligned with the objectives of the present study, and detailed mathematical definitions are provided in the Supplementary Materials.

(1) $$RMSE=\sqrt{\frac{1}{n}\sum_{i=1}^{n}{\left(y_{i}-{ŷ}_{i}\right)}^{2}}$$

(2) $$Normalized\;RMSE=\frac{RMSE}{max\left(y\right)-min\left(y\right)}$$

(3) $$PCC=\frac{Cov\left(y,ŷ\right)}{\sigma_{y}\cdot \sigma_{ŷ}}$$

(4) $$Custom\;Metric=0.5\times \left(1-NRMSE\right)+0.5\times PCC$$

### 3.4. Molecular Representations

In this study, four molecular representation methods were developed to comprehensively reflect the structural, physicochemical, and contextual information required to predict the CYP3A4 inhibition rates. First, ECFP8 (radius 4, 2048-bit) was employed to encode the presence or absence of substructures as binary fingerprint vectors. Next, RDKit [29] and Mordred [30] descriptors were employed to calculate physicochemical properties widely used in ADME analysis, including molecular weight, LogP, TPSA, hydrogen bond donor/acceptor counts, and rotatable bond counts.

The graph-based representation was constructed by modeling molecules as undirected atom–bond graphs, followed by integrating ring-level structural information through a dual-graph generation method. Node features included atomic number, formal charge, hybridization state, aromaticity, ring membership information, and the number of bondable hydrogens. Edge features included bond order, aromaticity, conjugation, and cyclicity. Additionally, to enable the GNN model to learn higher-order topological structural motifs, such as aromatic systems or fused rings, dual-graph tensors were incorporated, including ring masks, ring indices, and node–ring and ring–face (neighborhood) connectivity information. The generated dual-graph information was serialized into DGData objects and used as input for the O-GNN model [14].

To precisely map molecular structural information into a high-dimensional vector space, we adopted a dual-embedding strategy that integrates two pre-trained chemical language models (CLMs) with distinct architectures and training data distributions. To capture global structural information, we employed the MoLFormer-XL-both-10 pct [31] model, which is well suited for reflecting macroscopic structural factors—such as molecular size and overall shape—that influence CYP3A4 inhibitory activity, as well as for effectively modeling long-range dependencies within long SMILES sequences. In addition, to complement local chemical characteristics, we incorporated the Seyonec (PubChem10M_SMILES_BPE_450k) model trained on approximately 10 million PubChem compounds [32]. Mean pooling was applied to the final hidden states to extract substructure-level embeddings. Finally, the global and local embeddings obtained from the two models were concatenated to construct a unified molecular representation, which could provide robust and generalized predictive performance for targets with complex binding behaviors, such as CYP3A4.

### 3.5. Feature Engineering

Property engineering is structured on four axes. (i) Lipophilicity-polarity axis: FilterItLogS and TPSA were used to represent the balance between lipophilicity, polarity, and flexibility. (ii) Considering the electrostatic complementarity with CYP3A4’s heme and surrounding residues, indicators were constructed to represent the presence of positively or negatively charged patches and the relative size of the polar regions [1,3]. (iii) Functional Group/Structural Motif Axis: The presence/number of heterocyclic nitrogen motifs (e.g., imidazole, triazole, and pyridine), the ratio of metal-coordination-capable heteroatoms, and the aromatic carbon ratio were extracted to represent the heme coordination and interaction potentials [1,7]. (iv) Drug-like property axis: the number of violations of Lipinski’s rule of five and the QED score provide continuous and discrete indicators of overall drug-like properties [21]. The derived features were generated using the same structural normalization rules, serving as inputs for the ML model.

### 3.6. Data Augmentation

#### 3.6.1. Mixup Algorithm

**Figure 8:**
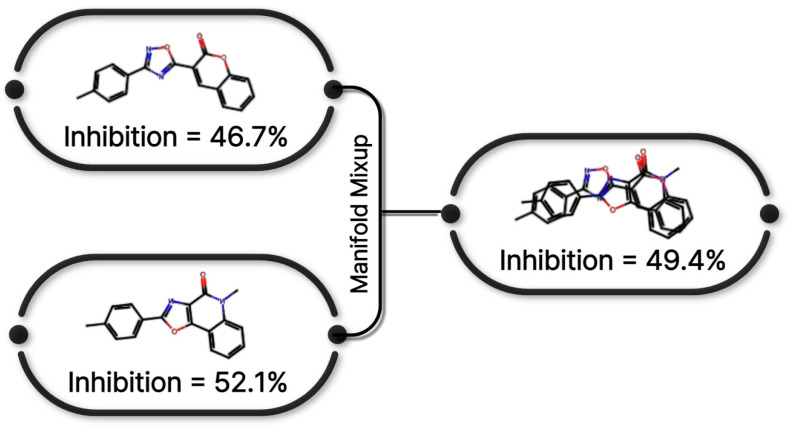
Manifold mixup strategy applied to O-GNN training. Interpolation is performed in the latent space using structurally and label-similar molecular pairs.

Figure 8 presents a conceptual illustration of the similarity-weighted manifold mixup strategy adopted in this study. Inspired by manifold mixup [16] and its extension to continuous regression settings [33], the proposed approach generates synthetic training samples by interpolating hidden representations of molecular graphs selected based on structural and label similarity.

Specifically, graph-level embeddings extracted from the O-GNN encoder were used to compute pairwise cosine similarity between molecular representations within each mini-batch. In parallel, label proximity was quantified based on the distance between CYP3A4 inhibition values. Candidate graph pairs were selected according to a composite similarity score, ensuring that interpolation occurred preferentially between structurally and functionally related compounds.

Formally, the structural similarity between two graph embeddings $h_{i}$ and $h_{j}$ was defined using cosine similarity:

(5) $$s_{g}\left(i,j\right)=\frac{h_{i}}{\left\|h_{i}\right\|}\cdot \frac{h_{j}}{\left\|h_{j}\right\|},\;\;s_{y}\left(i,j\right)=1-\frac{\left|y_{i}-y_{j}\right|}{R}$$

where $s_{g}(i,j)$ denotes the cosine similarity between the graph embeddings, and $s_{y}(i,j)$ represents the distance-based similarity between the corresponding CYP3A4 inhibition values. *R* is a normalization constant defined as the difference between the maximum and minimum CYP3A4 inhibition values in the dataset (5).

(6) $$S\left(i,j\right)=w_{g}s_{g}\left(i,j\right)+w_{y}s_{y}\left(i,j\right)$$

$w_{g},w_{y}\in \left[0,1\right]$ represents the weights for the graph and label similarities that satisfy $w_{g}+w_{y}=1$ . This composite similarity function is employed to determine the candidate pairs for the mixup (6).

(7) $$\tilde{h}=\lambda h_{i}+\left(1-\lambda \right)h_{j},\;ỹ=\lambda y_{i}+\left(1-\lambda \right)y_{j}$$

This formula generates new virtual samples through a linear interpolation in the embedding space, enabling the model to learn both structural diversity and continuous distributions (7).

(8) $$\lambda \sim Beta\left(\alpha ,\alpha \right)$$

*λ* is the mixing ratio sampled from a Beta distribution. The interpolation coefficient *λ* probabilistically controls the interpolation ratio between samples and encourages diversity in the generated representations (8).

Importantly, the interpolated representations were forwarded exclusively to the prediction head, enabling manifold mixup within the hidden layer while preserving the original graph encoder structure. All mixup operations were restricted to the training folds to prevent information leakage. This similarity-weighted interpolation scheme generates meaningful synthetic samples that are structurally coherent and consistent with target inhibition values, thereby improving model smoothness and generalization performance in continuous label spaces [16,33].

#### 3.6.2. Test-Time Augmentation

**Figure 9:**
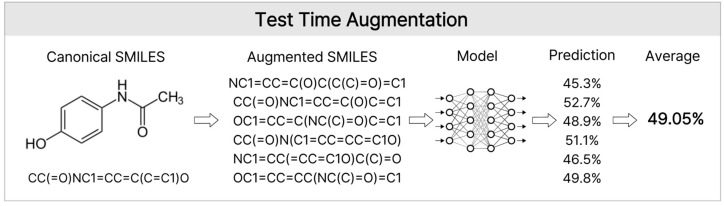
Test Time Augmentation. This diagram shows the TTA process used during inference. A single molecule was converted into multiple Randomized SMILES strings along with its Canonical SMILES These diverse representations were processed by the model, and the resulting predictions were averaged (mean pooling) to produce a robust final prediction. This approach mitigates the bias emerging from specific SMILES serializations.

TTA generates different input formats for a single molecule, obtains predictions for each molecule, and averages them to produce a final prediction. In this study, the TTA was implemented by leveraging the diversity of the SMILES notation [34]. As the same molecule can be represented by multiple SMILES strings, we generated one Canonical SMILES and N Randomized SMILES for each compound. Canonical SMILES provide standardized structural information, whereas Randomized SMILES randomly reorders the atoms to generate diverse representations of the same structure. All generated SMILES strings were independently processed using the MoLFormer (MoLFormer-XL-both-10 pct) and Seyonec (PubChem10M_SMILES_BPE_450k) models to obtain the corresponding predictions. Subsequently, the predicted values for all augmented samples sharing the same MolID were averaged (TTA mean pooling) and used as the final predicted values for that molecule (Figure 9). This SMILES-based TTA procedure minimizes bias from a single notation system and enables more stable and generalized predictions by integrating diverse representative information about the molecular structure.

### 3.7. Models and Training

The proposed framework consists of three main components: (1) a deep learning block based on O-GNN with contrastive learning and mixup, (2) a vector-based ML block incorporating LightGBM, XGBoost, and FastAI models, and (3) a TTA module. The vector-based ML block employs tree-based and nonlinear learning models, which are relatively robust to correlated input features.

Outputs from these three components were integrated using a weighted ensemble strategy to generate the final prediction. Specifically, the final % inhibition rate was obtained by combining predictions from the deep learning (GNN), vector-based models (LightGBM, XGBoost, and NeuralNetFastAI [35]), and SMILES enumeration-based TTA components based on their respective contributions.

Deep Learning (GNN). For the graph-based prediction model, we adopted O-GNN [14], a modified GNN that explicitly models rings as independent third structural units, in addition to atoms (nodes) and bonds (edges) in the existing molecular graph. Each layer follows a sequential structure: (i) updating the bond representations, (ii) updating the atom representations, and (iii) updating the ring representations. Message passing combines self-attention and the MLP to maximize nonlinearity. The node update step reflects the aggregated values of adjacent nodes and edges while integrating the (weighted) average of all ring representations to which the node belongs. This design ensures that information, including aromaticity, ring environment, and fused rings, is not lost during the readout process. This structure addresses the weaknesses of existing MPNNs, which struggle to distinguish ring structures sufficiently owing to their depth × width limitations. It exhibits high expressive power, enabling the identification of isomorphic subgraphs located on various rings, even within a single layer [14].

In the pre-labeling learning stage, InfoNCE-based contrastive learning was introduced. Maximizing the representational similarity of positive pairs (composed of augmented views of the same molecule, such as atomic masking, bond deletion, or subgraph removal) and ensuring sufficient separation from negative pairs (different molecules) enhance the structural generalization performance even in environments with scaffold bias [15]. Similarly, MetaboGNN significantly improves metabolic stability prediction performance through contrastive learning [36].

A manifold mixup was applied during the fine-tuning stage [16]. We selected sample pairs based on the weighted sum of the cosine similarity between graph embeddings and label similarity within a batch. Subsequently, we linearly interpolated the hidden embeddings and continuous labels to generate structurally valid synthetic samples that appeared natural in the label space [33]. All mixup operations were confined to the folds to prevent data leakage. The GNN block, structured by combining mixing with a contrastive-learning-based pre-trained O-GNN, underwent 5-fold cross-validation and was trained for 100 epochs per fold.

Machine Learning (vector-based). Vector-based models comprised LightGBM, XGBoost, and NeuralNetFastAI (tabular MLP). The input features consisted of a single vector-based representation integrating ECFP8, RDKit, and Mordred molecular descriptors, and the derived features generated through feature engineering. Each model was subjected to a continuous % inhibition rate regression. Although NeuralNet-FastAI is a deep learning-based architecture, it belongs to the vector input pipeline rather than the graph input pipeline; thus, it was classified as an ML block in this study.

TTA. For each molecule, Canonical SMILES and N randomized SMILES were generated and applied to the pre-trained CLMs MoLFormer-XL-both-10 pct and Seyonec (PubChem10M_SMILES_BPE_450k), respectively. Multiple predictions obtained for the same molecule were arithmetically averaged and integrated with the mean TTA value. This mitigates the representation bias emerging from the SMILES notation order and enumeration schemes, effectively reducing variance during inference.

**Figure 10:**
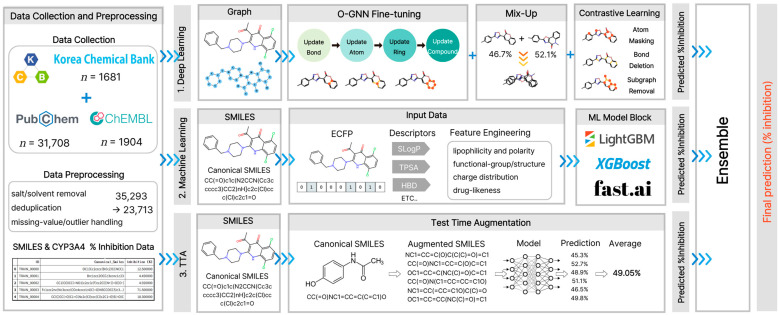
Overall architecture of the proposed hybrid ensemble model. Predictions from machine learning models, GNNs, and test-time augmentation are combined to generate the final CYP3A4 inhibition prediction.

Final Ensemble. In the intra-model stage, the TTA average predictions from each base model (O-GNN, XGBoost, LightGBM, and NeuralNetFastAI) were aggregated into single values. At the inter-model stage, these values were combined using a weight-based ensemble to calculate the final % inhibition rate. This dual-ensemble approach combines the strengths of graph-based structure learning with the complementarity of a vector-based nonlinear approximation, simultaneously achieving variance reduction and bias correction compared with a single model (Figure 10).

### 3.8. Software and Hardware Specifications

The computational experiments were conducted on a Linux-based high-performance computing system (Linux-6.6.105; Google LLC, Mountain View, CA, USA) equipped with an NVIDIA A100-SXM4-80GB GPU (NVIDIA Corporation, Santa Clara, CA, USA). The environment was built on Python (v3.12.12). Deep learning models were implemented using PyTorch (v2.2.0+cu118), PyTorch Geometric (v2.5.0), and DGL (v2.1.0). Molecular descriptors and graph structures were processed using mordred (v1.2.0), RDKit (v2025.09.3), and networkx (v2.8.8). Machine learning models were optimized via AutoGluon (v1.3.1), LightGBM (v4.6.0), XGBoost (v3.0.5), CatBoost (v1.2.8), and Scikit-learn (v1.6.1). Model interpretability was analyzed using SHAP (v0.48.0).

## 4. Conclusions

In this study, we developed a hybrid AI framework that integrates vector-based ML and GNNs to predict CYP3A4% inhibition with high accuracy and robustness. By curating and harmonizing 23,713 inhibition–structure pairs from the KCB [25] and public databases, including ChEMBL [26] and PubChem [27], and by combining ECFP8 fingerprints [24], RDKit [29]/Mordred [30] descriptors, dual-graph molecular representations, and SMILES-based language model embeddings, the proposed system captured both global physicochemical properties and local graph-level patterns relevant to metabolic liability.

The final ensemble, which combined a LightGBM-based [11] ML block with an O-GNN [14] model fine-tuned using contrastive learning [15] and manifold mixup [16], and further stabilized by SMILES test-time augmentation [34], achieved an RMSE of 19.0784 and a PCC of 0.7570 on the internal validation set, as well as a Custom Metric of 0.8035 on an independent external dataset. These results demonstrate that the hybrid approach can provide stable and quantitatively reliable predictions of CYP3A4 inhibition by chemically diverse compounds.

Analysis of feature importance, correlation patterns, and SHAP-based substructure contributions consistently highlighted molecular size, hydrophobicity, polarizability, and aromatic heterocycles, such as benzodioxole, imidazole, and pyridine, as key determinants of CYP3A4 inhibition, while certain saturated motifs, including piperidine, were associated with reduced activity. These insights are consistent with the known structural characteristics of the CYP3A4 active site [3,4] and provide practical guidance for the design and prioritization of new chemical entities.

However, the current model is limited to a single enzyme (CYP3A4) and relies on heterogeneous % inhibition data generated under varying experimental conditions. Future work will extend the framework to multi-task prediction across multiple CYP isoforms and prospective validation and will incorporate protein structure-aware representations to further enhance predictive performance and broaden its applicability in drug discovery. Furthermore, we aim to leverage this framework as a generalizable backbone for other ADMET-related endpoints, including the development of toxicity prediction models and related applications.

## Figures and Tables

**Table 1:** Performance Comparison of Machine Learning, Graph Neural Networks, and the Proposed Hybrid GNN-ML Model.

Category	Model	RMSE	R2	PCC	Custom Metric
ML	CatBoost	19.9161	0.5343	0.7313	0.7661
ML	XGBoost	19.9099	0.5345	0.7326	0.7668
ML	LightGBM	19.7346	0.5427	0.7375	0.7701
ML	Weighted Ensemble	19.1031	0.5715	0.7566	0.7828
GNN	GAT	20.9954	0.4824	0.6960	0.7430
GNN	D-MPNN	20.9835	0.4829	0.6963	0.7432
GNN	GINE	20.3554	0.5135	0.7204	0.7584
	O-GNN				
GNN	Contrastive Learning	20.1002	0.5257	0.7265	0.7627
	Mixup				
	Weighted Ensemble				
GNN-ML	O-GNN	**19.0784**	**0.5726**	**0.7570**	**0.7831**
	Test Time Augmentation				

**Table 2:** Top Physicochemical Features Positively Correlated with CYP3A4 Inhibitory Activity.

Feature	Spearman ρ
Molar Refractivity	+0.444
MW	+0.411
SLogP	+0.411
Aromatic Rings	+0.404

## Data Availability

The original contributions presented in this study are included in the article/Supplementary Material. Further inquiries can be directed to the corresponding author.

## Supplementary Materials

The following supporting information can be downloaded at: https://github.com/Sominuu/CYP3A4-Inhibition-prediction/tree/main (accessed on 27 January 2026).
